# Comparing emotions in ChatGPT answers and human answers to the coding questions on Stack Overflow

**DOI:** 10.3389/frai.2024.1393903

**Published:** 2024-09-16

**Authors:** Somayeh Fatahi, Julita Vassileva, Chanchal K. Roy

**Affiliations:** Department of Computer Science, University of Saskatchewan, Saskatoon, SK, Canada

**Keywords:** generative AI, large language models, natural language processing, emotion analysis, Stack Overflow, ChatGPT, programming assistance

## Abstract

**Introduction:**

Recent advances in generative Artificial Intelligence (AI) and Natural Language Processing (NLP) have led to the development of Large Language Models (LLMs) and AI-powered chatbots like ChatGPT, which have numerous practical applications. Notably, these models assist programmers with coding queries, debugging, solution suggestions, and providing guidance on software development tasks. Despite known issues with the accuracy of ChatGPT’s responses, its comprehensive and articulate language continues to attract frequent use. This indicates potential for ChatGPT to support educators and serve as a virtual tutor for students.

**Methods:**

To explore this potential, we conducted a comprehensive analysis comparing the emotional content in responses from ChatGPT and human answers to 2000 questions sourced from Stack Overflow (SO). The emotional aspects of the answers were examined to understand how the emotional tone of AI responses compares to that of human responses.

**Results:**

Our analysis revealed that ChatGPT’s answers are generally more positive compared to human responses. In contrast, human answers often exhibit emotions such as anger and disgust. Significant differences were observed in emotional expressions between ChatGPT and human responses, particularly in the emotions of anger, disgust, and joy. Human responses displayed a broader emotional spectrum compared to ChatGPT, suggesting greater emotional variability among humans.

**Discussion:**

The findings highlight a distinct emotional divergence between ChatGPT and human responses, with ChatGPT exhibiting a more uniformly positive tone and humans displaying a wider range of emotions. This variance underscores the need for further research into the role of emotional content in AI and human interactions, particularly in educational contexts where emotional nuances can impact learning and communication.

## 1 Introduction

With the advancement of technology, especially in artificial intelligence (AI), we are witnessing the emergence of novel tools. Over the past decade, text-based chatbots have gained widespread popularity across diverse application domains. This surge in adoption has been described as a ‘chatbot tsunami’ (Grudin and Jacques, 2019), enabling human interaction with machines through natural written language. In November of 2022, OpenAI introduced ChatGPT-3.5 (Open AI, 2023), a chatbot AI built on top of existing Large Language Models (LLMs) to facilitate interactive communication through a conversational interface. OpenAI achieved this interactive capability by employing reinforcement learning from human feedback, building upon prior work from InstructGPT (Ouyang et al., 2022). ChatGPT rapidly gained popularity and attained a milestone by amassing 100 million users by January 2023 (Saini, 2023), reaching 1.5 billion monthly visitors as of the time of writing this paper. ChatGPT can generate diverse text forms, encompassing scientific abstracts, domain-specific question answers, programming code, lifelike conversational exchanges, text summarization, language translation, and providing suggestions and recommendations. However, ChatGPT also carries potential risks, such as enabling copyright violations, plagiarism, over-dependence, and possibly reduced creativity. Also, studies indicate that people are concerned about cybersecurity threats posed by malicious entities using ChatGPT to create harmful code, hack, gather information, and trick people into revealing private or sensitive information (Okey et al., 2023; Khoury et al., 2023; Poremba, 2023; Malwarebytes, 2023). The risk of AI-generated content being passed off as human-written may lead to potential harm, such as the spread of fake content on social media. AI-generated content is riskier than human-written posts because AI can produce vast amounts of tailored misinformation quickly, making it hard to detect and flag. Its ability to personalize messages increases their persuasiveness, and the autonomous, adaptable nature of AI allows it to continuously evolve and evade detection. It could also cause significant problems in various areas, such as information security and digital forensics. In addition to its ability to provide specific answers to user questions, ChatGPT can be utilized for completing written assignments and examinations on behalf of students, raising concerns about AI-assisted cheating (Susnjak and McIntosh, 2024). In response, some schools have implemented bans on access to ChatGPT on campus (Dibble, 2023). The implications of ChatGPT in the field of education were explored in a review and the findings revealed that educators expressed concerns about the use of ChatGPT in education, fearing that students might outsource their work to ChatGPT due to its capability to rapidly generate acceptable texts (Mhlanga, 2023). Although the development of ChatGPT can be challenging, it may simplify the application of AI in teaching and learning, making it more accessible for instructors and helping students increase their knowledge in a proper way. Despite the drawbacks associated with misusing AI Chatbots like ChatGPT, there are numerous advantages in its application in education. These include personalized tutoring: ChatGPT facilitates personalized tutoring, leading to enhanced learning outcomes (Alshahrani, 2023). Automated essay grading: It streamlines the essay grading process, saving valuable time for teachers (Parker et al., 2023). ChatGPT aids in language translation, making educational materials more accessible to a broader audience (Zhao et al., 2024). Interactive learning: It promotes interactive learning, offering effective support for students (Murad et al., 2023). Adaptive learning: ChatGPT could potentially adapt teaching methods based on a student’s progress and performance, especially in the context of learning programming languages.

Given the significant influence of emotions on perception, the learning process (Tyng et al., 2017), and communication in humans, there is substantial evidence indicating that learning is intertwined with emotions (Hökkä et al., 2020; Bohn-Gettler and Kaakinen, 2022; Um et al., 2012). Consequently, if ChatGPT interacts with humans in a way that aligns with the positive aspects such as optimism and positive emotions, it could prove more beneficial in the field of education by effectively assuming the role of a teacher, as previous research has shown the impact of AI on learning (Wang et al., 2024; Kasneci et al., 2023). However, there is still a lack of research in education comparing the emotional content in human and AI responses, which warrants further investigation for a comprehensive understanding.

The current trend in chatbot development is toward empathetic and emotionally intelligent bots, capable of recognizing user feelings and generating fitting answers (Adamopoulou and Moussiades, 2020). However, to what extent can AI chatbots understand human emotions and respond with a human level of empathy, and to what extent can they mimic believable emotional responses to a situation? Despite notable advancements in chatbot development (Adam et al., 2021; Rapp et al., 2021; Adamopoulou and Moussiades, 2020), accurately capturing and expressing the right emotions within chatbot interactions remains a persistent challenge. ChatGPT has shifted researchers’ perspectives to some extent. For instance, one study (Elyoseph et al., 2023) showcased ChatGPT’s capacity to produce suitable Emotional Awareness (EA) responses, with the potential for significant improvement over time.

However, there is a gap in research when it comes to comparing the emotional aspects of human and ChatGPT responses in different areas. Our study focuses on understanding these differences in how questions related to programming language are answered. Learning programming is tough for many in education today, and ChatGPT can assist students and programmers with problem-solving. That is why we have chosen this field to look into the emotional differences in responses between humans and ChatGPT. The findings from this research may offer useful insights that could inform future developments of ChatGPT, enhancing its utility for education and learning.

To accomplish this goal, we address the following research questions:

**Research Questions**:

**RQ1**: What is the distribution of emotions in questions, human-generated answers and ChatGPT answers?

**RQ2**: What are the dominant emotions exhibited in ChatGPT answers, and what are the dominant emotions in human-generated answers?

**RQ3**: How does the range of expressed emotions differ between human generated and ChatGPT answers?

To answer our research questions, we conducted a comprehensive analysis, comparing emotional aspects in responses from both ChatGPT and humans to 2000 questions sourced from Stack Overflow (SO).

The subsequent sections of this paper are structured as follows: Section 2 delves into the literature review of our study, providing an overview of recent research related to ChatGPT. Section 3 outlines our methodology. The results are presented in Section 4, followed by the discussion in Section 5 and the conclusion in Section 6.

## 2 Literature review

Since November 2022, ChatGPT has attracted significant attention, resulting in numerous applications and extensive research (Kalla et al., 2023). This section aims to provide a clearer introduction to the applications of ChatGPT within the context of software engineering. Specifically, we explore its utility within the Stack Overflow dataset, focusing on studies that examine ChatGPT’s role and effectiveness in this domain.

Jalil et al. (2023), the researchers assess the performance of ChatGPT in addressing common queries from a used software testing curriculum. Their investigation shows that ChatGPT’s current capabilities effectively address 77.5% of the questions examined. Among these questions, it provides fully or partially accurate answers in 55.6% and correct or partially correct explanations in 53% of the cases. The findings of this research diverge from the outcomes of implementing ChatGPT in other fields, such as medicine (Kung et al., 2023) or law (Choi et al., 2021), where ChatGPT has demonstrated success in passing specific portions of their examinations. This discrepancy suggests that although ChatGPT exhibits capability, it also possesses limitations, including a lack of comprehensive knowledge and the tendency to make incorrect assumptions, contributing to potential response inaccuracies.

Surameery and Shakor (2023), researchers investigated the role of ChatGPT in solving programming bugs. They found that ChatGPT is superior to other tools in cost, speed, customization, ease of use, and scalability. However, when it comes to fitting into existing systems, traditional debugging tools are more effective due to their integration capabilities. Additionally, the accuracy of ChatGPT depends on the quality of its training data, while traditional debugging tools generally provide higher accuracy levels.

An empirical investigation (Nascimento et al., 2023) compared the performance of software engineers and AI systems, such as ChatGPT, across various evaluation metrics. ChatGPT-generated code was evaluated against code created by developers and submitted on Leetcode. The study revealed that automated systems like ChatGPT can, in certain instances, surpass the performance of novice software engineers in specific tasks. This superiority was particularly apparent in resolving easy and medium-level problems, where ChatGPT consistently outperformed novice contest programmers.

Kabir et al. (2023), a comprehensive analysis was conducted on ChatGPT’s answers to 517 questions sourced from Stack Overflow (SO). The assessment encompassed the correctness, consistency, comprehensiveness, and conciseness of these answers. The manual analysis indicated that 52% of the answers provided by ChatGPT contained inaccuracies, while 77% were found to be excessively verbose. Nevertheless, users preferred ChatGPT’s answers 39.34% of the time due to their thoroughness and articulate language style. The results of the linguistic analysis demonstrated the formal nature of ChatGPT’s answers, which rarely expressed negative sentiments. Although the user study showed that users had a higher preference and quality rating for SO, they occasionally erred by favoring incorrect ChatGPT answers due to the model’s well-articulated language style and seemingly plausible logic presented with positive assertions. These findings highlight the requirement for meticulous error correction within ChatGPT while also emphasizing the need to make users aware of the potential risks associated with answers that appear accurate.

Liu et al. (2023) conducted a study to examine the comparative efficacy of ChatGPT and SO in assisting programmers. Two groups of students with similar programming abilities were instructed to use the two platforms to solve three programming tasks: algorithmic challenges, library usage, and debugging. The findings reveal that, in terms of code quality, ChatGPT exhibits significantly better performance than SO when aiding in the completion of algorithmic and library-related tasks. However, Stack Overflow proves more beneficial for debugging tasks. Concerning task completion speed, the ChatGPT group demonstrates notably faster results than the SO group, specifically in algorithmic challenges, while displaying similar performance in the other two tasks.

Delile et al. (2023) explore the privacy issues encountered by developers. They compare the responses accepted on SO with those generated by ChatGPT for these queries to evaluate if ChatGPT could be a helpful alternative. The results reveal that most privacy-related questions center on choice/consent, aggregation, and identification. Additionally, ChatGPT provides roughly 56% of responses that match the accuracy level of SO.

Following Stack Overflow’s decision to ban ChatGPT, Borwankar et al. (2023) examined how the users of SO responded to this change. They studied the quality of content using natural language processing (NLP) techniques and voting patterns across SO and the AskProgramming subreddit on Reddit. The results indicate that SO users adjusted their answer style after the limitation, leading to more positive, longer responses than AskProgramming subreddit users. This study shows that there has been an improvement in content quality post-limitation, reflected in increased upvotes for answers.

The research discussed in this section sheds light on various aspects of ChatGPT’s capabilities and limitations in software engineering. A prevailing consensus suggests that the ability to differentiate between ChatGPT and human-generated text is crucial. As mentioned, emotion is a distinguishing factor for identifying the human and ChatGPT (Pamungkas, 2019). However, it is noteworthy that relatively few studies have investigated emotion in communication with ChatGPT. Elyoseph et al. (2023) focused on assessing the emotional awareness (EA) capabilities of ChatGPT. Using the Levels of Emotional Awareness Scale (LEAS), researchers conducted two examinations involving 20 scenarios to evaluate ChatGPT’s EA performance, comparing it to norms established by a previous study. ChatGPT’s emotional awareness (EA) scores were compared to those in a previous study (Nandrino et al., 2013). To check how accurate ChatGPT’s answers were, two professional psychologists independently rated each answer based on how well it fits the situation. They used a scale from 0, meaning “the feelings described do not match the scenario at all,” to 10, meaning “the emotions described fit the scenario perfectly.” The results illustrated that ChatGPT can produce suitable emotional awareness (EA) answers, potentially enhancing performance over time.

To the best of our knowledge, no existing research compares emotions in answers generated by ChatGPT with those created by humans in the software engineering domain. Additionally, there has yet to be a study focused on using emotion as a fingerprint to differentiate between human and ChatGPT-generated answers. This paper addresses these gaps and delves into this unexplored research area.

## 3 Methodology

This study uses a comparative analysis approach to assess the answers generated by ChatGPT in contrast to those provided by humans on Stack Overflow.

### 3.1 Data

We chose the subject matter, Software Engineering, because it is less inclined toward emotional interactions, as it is primarily about resolving programming problems. This makes it a more ‘neutral’ domain for analysis. For instance, datasets containing reviews of products, hotels, or restaurants are likely to feature numerous strongly opinionated positive and negative reviews. This could skew the comparison, placing a heavier emphasis on human emotions.

As a source of data, we chose Stack Overflow because it is a popular free question-and-answer community, extensively used for many years by programmers and software engineers (Spolsky and Atwood, 2008), and the data is readily accessible and abundant. We used the open dataset^1^ provided in Kabir et al., 2023, comprising answers from ChatGPT and human experts to a randomly selected set of 2000 Stack Overflow questions. As an example, one of the data points is presented in Table 1.

**Table 1:** A sample of Stack Overflow questions with answers from human and ChatGPT.

Question	I need to grab the base64-encoded representation of the ViewState. Obviously, this would not be available until fairly late in the request lifecycle, which is OK. For example, if the output of the page includes: <input type=“hidden”name=“__VIEWSTATE” id=“__VIEWSTATE” value=“/wEPDwUJODU0Njc5MD…==” /> I need a way on the server side to get the value “/wEPDwUJODU0Njc5MD…==” To clarify, I need this value when the page is being rendered, not on PostBack. e.g. I need to know the ViewState value that is being sent to the client, not the ViewState I’m getting back from them.	Human answer	Rex, I suspect a good place to start looking is solutions that compress the ViewState—grabbing ViewState on the server before it is sent down to the client and gzipping it. That is exactly where you want to be. Scott Hanselman on ViewState Compression (2005) ViewState Compression with System.IO.Compression (2007)
		ChatGPT answer	To get the base64-encoded representation of the ViewState on the server side, you can use the following code in your ASP.NET page: “csharp string viewStateValue = Page.ClientScript.GetHiddenField(‘__VIEWSTATE’).Value This code uses the ‘ClientScript’ object to access the hidden ‘__VIEWSTATE’ field and retrieve its value”. Note that this code should be executed after the ViewState has been populated, which typically happens during the Page’s “PreRender” event. You can add the code in the appropriate event handler of your page, such as the “Page_Load” or “Page_PreRender” event. Keep in mind that the ViewState value may not be available until the page has been fully rendered, so you might need to experiment with different events to find the right timing for retrieving it

### 3.2 Model

To extract emotions from the questions, ChatGPT answers, and human answers, we utilized an emotion-multilabel model from Hugging Face. This model is a fine-tuned version of cardiffnlp/twitter-roberta-base-2022-154 m (Mohammad et al., 2018) and is based on EmoBERT, a novel emotion-based variant of the BERT (Devlin et al., 2018) transformer model. Multi-label EmoBERT comprises three main components. The first component involves two encoders: one for all tokens (Word Encoder) and another designed explicitly for emotion-word hashtag tokens (Hashtag Encoder). The second component is a compositional layer that represents sentiment semantic composition. The final component is a label correlation layer that learns the correlation between co-existing emotions.

The emotions were selected for this model based on the basic emotion model (Ekman, 1992; Plutchik, 1980; Parrott, 2001; Frijda, 2017) and the valence–arousal–dominance (VAD) model (Russell, 1980). Finally, researchers considered an emotion classification task encompassing 11 emotions commonly expressed in tweets: anger, anticipation, disgust, fear, joy, love, optimism, pessimism, sadness, surprise, and trust for this model.

**Figure 1:**
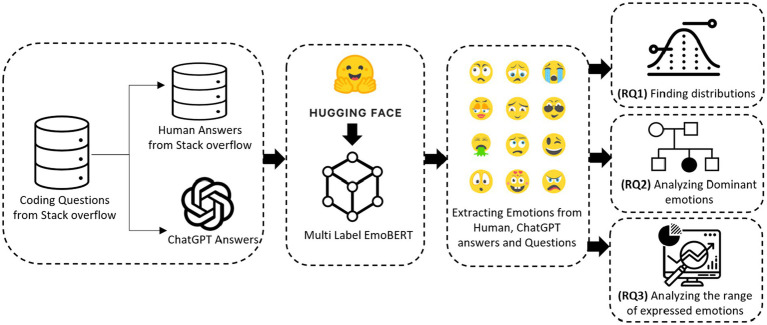
Schematic diagram of our study.

Its performance demonstrated close approximation to published results in extracting emotions from the Stack Overflow dataset, achieving a Micro-F1 score of 83.36 (Li and Xiao, 2023). The schematic diagram of our study is shown in Figure 1.

## 4 Results

**Figure 2:**
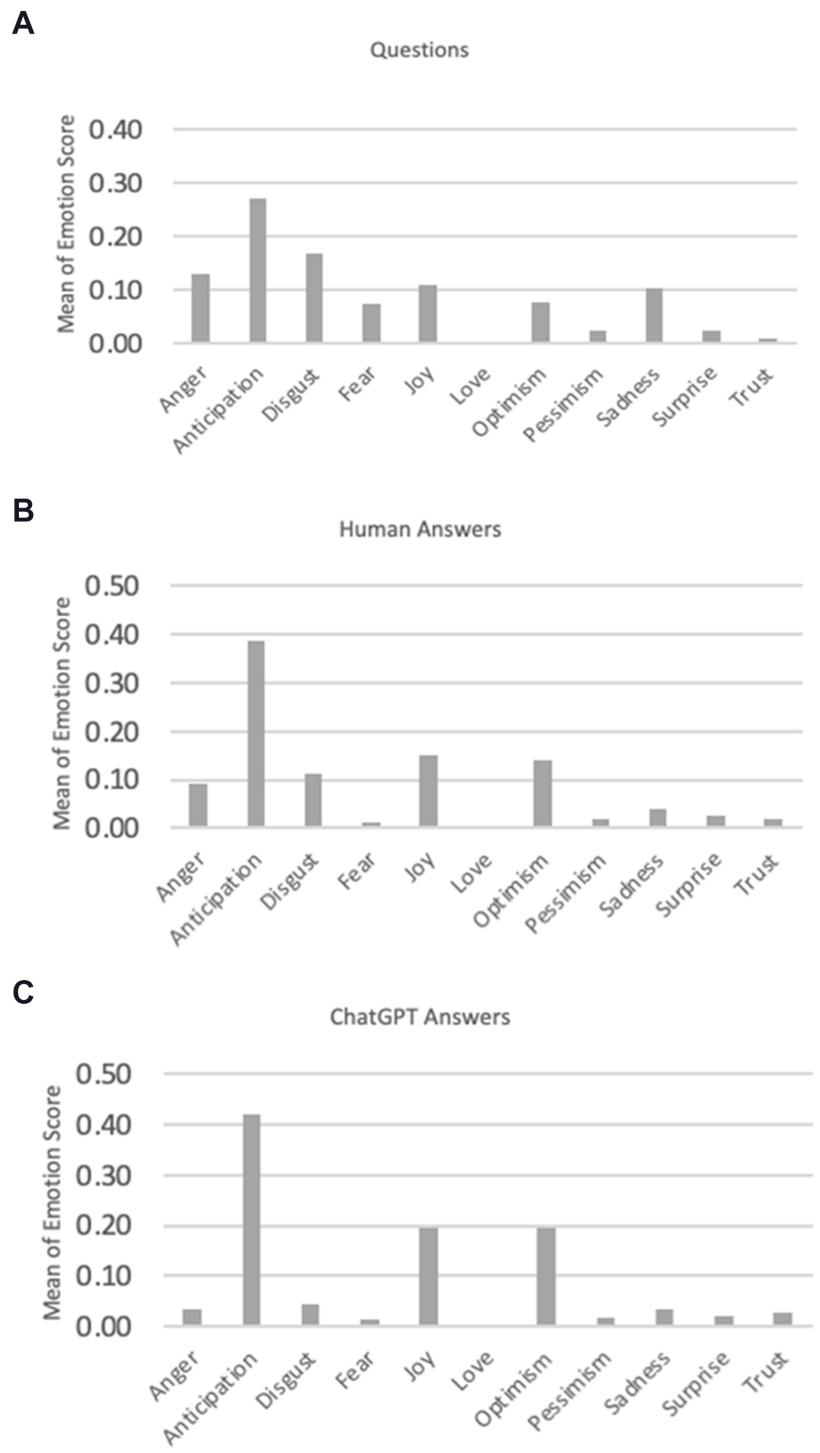
Means of emotion scores for SO questions, human answers, and ChatGPT answers.

When the Multi-label EmoBERT model is applied to the dataset, it produces a vector of emotions as an output (Table 2). This vector comprehensively represents the emotional content embedded within the questions and the sets of humans and ChatGPT answers. In the initial phase, we used the Chi-squared test to examine the relationship between the emotion in the question and the emotional response from both humans and ChatGPT. As depicted in Table 3, our findings indicate a notable correlation between the emotions conveyed in questions and the emotional responses from both human participants and ChatGPT. It should be mentioned that we opted to use the Chi-squared test instead of the Pearson correlation for the following reasons. The Chi-squared test is particularly suitable for categorical data, in contrast, the Pearson correlation coefficient is designed to measure the linear relationship between two continuous variables. Our data consisted of categorical variables in this step, making the Chi-squared test the appropriate choice. Also, the Chi-squared test is used to determine whether there is a significant association between two categorical variables. Our objective was to assess the independence or association between these variables. The Pearson correlation assumes a linear relationship between variables and can provide misleading results when applied to non-linear or non-continuous data. The Chi-squared test does not require any assumptions about the nature of the relationship between the variables other than their categorical nature, making it more flexible and appropriate for our analysis. In the next step, we calculate the average value of emotions in SO questions, human answers, and ChatGPT answers. As Figure 2 shows, questions frequently exhibit emotions such as anticipation, anger, and disgust. There is a notable similarity in emotional patterns between humans and ChatGPT.

**Table 2:** Values of emotions for a sample of Stack Overflow questions and answers.

Emotions	Anger	Anticipation	Disgust	Fear	Joy	Love	Optimism	Pessimism	Sadness	Surprise	Trust
SO Question	0.03	0.72	0.04	0.01	0.05	0	0.06	0.02	0.02	0.04	0.02
Human Answer	0.11	0.45	0.12	0.01	0.13	0.01	0.05	0.02	0.04	0.06	0.02
ChatGPT Answer	0.02	0.49	0.02	0	0.24	0.01	0.15	0.01	0.01	0.02	0.03

**Table 3:** Relationship between emotions in Stack Overflow questions and answers from human and ChatGPT.

Emotion in Stack Overflow and Human answer	*X^2^* (df = 10, *N* = 2000) = 123.74, *p* < 0.001
Emotion in Stack Overflow and ChatGPT answer	*X^2^* (df = 10, *N* = 2000) = 311.32, *p* < 0.001

Upon a detailed comparison of human and ChatGPT answers, shown in Figure 2, it becomes evident that ChatGPT answers tend to be more optimistic and joyful, while human answers often contain more expressions of anger and disgust.

**Figure 3:**
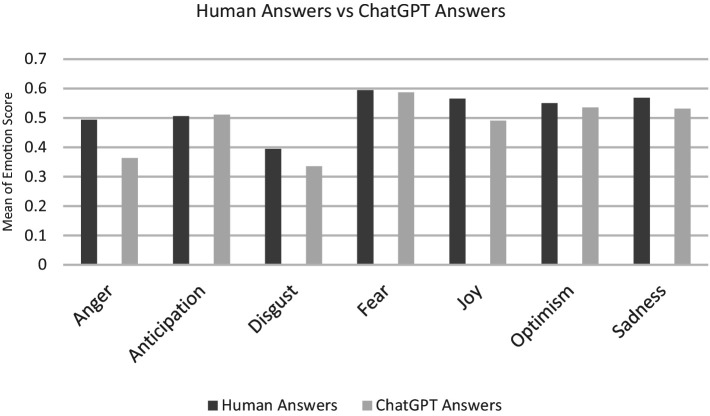
Comparison of dominant emotions in human answers and ChatGPT answers.

In the next step, our focus shifts to identifying the predominant emotion in both the questions and their corresponding answers. We determine the maximum emotion intensity for each answer rather than considering a range of different emotions. As shown in Table 2, a spectrum of emotions is present, but Anticipation emerges as the dominant emotion for both the question and the answers from humans and ChatGPT. Now we focus our attention on the dominant emotions, excluding those detected marginally, e.g., Love, Pessimism, and Trust (see Figure 3). Notably, when we focus solely on the dominant emotion for each question/answer and calculate the mean emotion value, it differs from the previous calculation.

**Figure 4:**
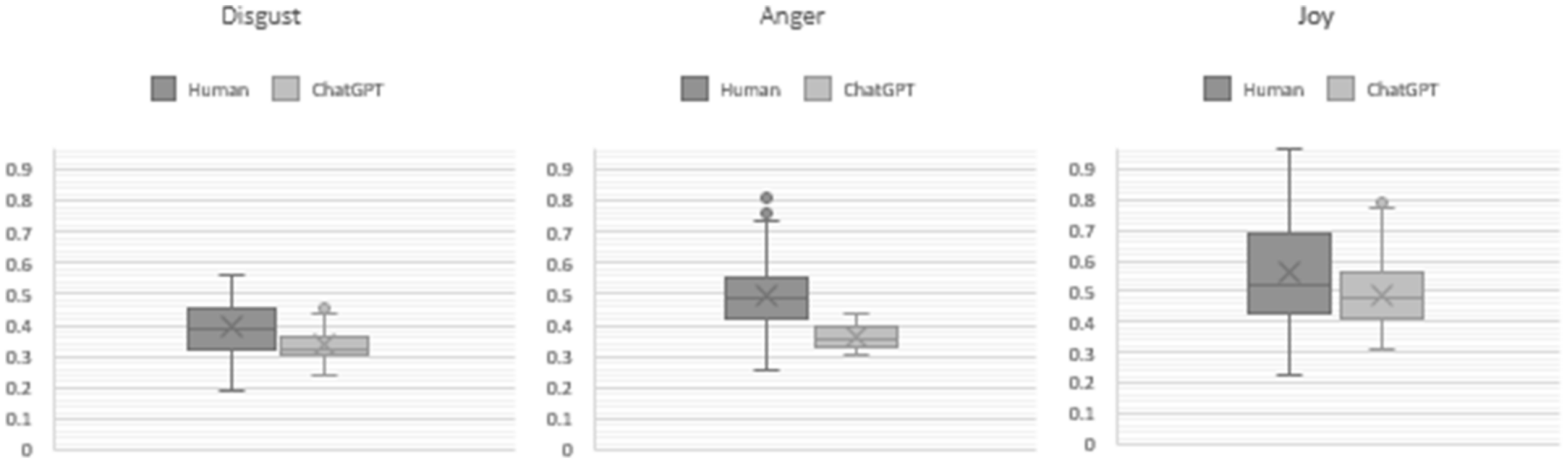
Box plot comparison of emotional responses between ChatGPT and human.

To understand the data distribution, we examined how the data is dispersed using a box plot (Figure 4). The results show that for some emotions, such as Anger, Disgust, and Joy, there are considerable differences between ChatGPT and human responses. Notably, human emotions exhibited a wide dispersion. Conversely, for emotions like Anticipation, Optimism, and Sadness, the distributions appeared consistent between human and ChatGPT-generated answers.

We performed a t-test to compare the data represented in the boxplots shown in Figure 4. For the “Anger” variable, the results indicate that the t-statistic is 6.27 and the *p*-value is 1.28e-6. These results suggest a highly significant difference between the groups being compared. The large t-statistic indicates a substantial difference in the means of the two groups, while the extremely small p-value indicates that this difference is statistically significant, far below the conventional significance level of 0.05. This provides strong evidence that the observed differences in “Anger” levels between the groups are not due to random chance.

For “Disgust,” the results show that the t-statistic is 4.36 and the p-value is 1.89e-5. Since the p-value (0.0000189) is far below the common significance level of 0.05, we can reject the null hypothesis. This means there is strong evidence to suggest a significant difference between the means of the two groups.

For “Joy,” the p-value is 4.41e-7 (0.00000044), which is much smaller than the conventional significance level of 0.05. This indicates that there is a statistically significant difference between the two groups.

In the next step, we applied topic modeling (Alghamdi and Alfalqi, 2015) to uncover the relationships between topics and emotions (Table 4). Specifically, we utilized Latent Dirichlet Allocation (LDA; Blei et al., 2003), a probabilistic generative model widely employed in natural language processing (NLP) and machine learning. LDA is a technique adept at identifying underlying topics within a set of documents. In our analysis, we employed the LDA model on the questions, human answers, and ChatGPT-generated answers to extract topics.

**Table 4:** Topic modeling results.

Topic Label	Top 10 words in the topic
Text Processing and Data Structures	emacs, dictionary, date, printf, echo, hello, struct, sizeof, iphone, margin
File and Project Management	file, path, files, server, folder, directory, project, command, version, windows
Application Development and Performance	code, performance, like, tools, application, http, provides, data, specific, good
Database Operations and User Management	table, data, query, code, thread, database, user, session, server, page
Visual Design and Libraries	color, myclass, bean, boost, colors, datetime, signal, iterator, serialization, vector
Object-Oriented Programming and Methods	string, class, function, value, public, code, method, return, event, object

**Figure 5:**
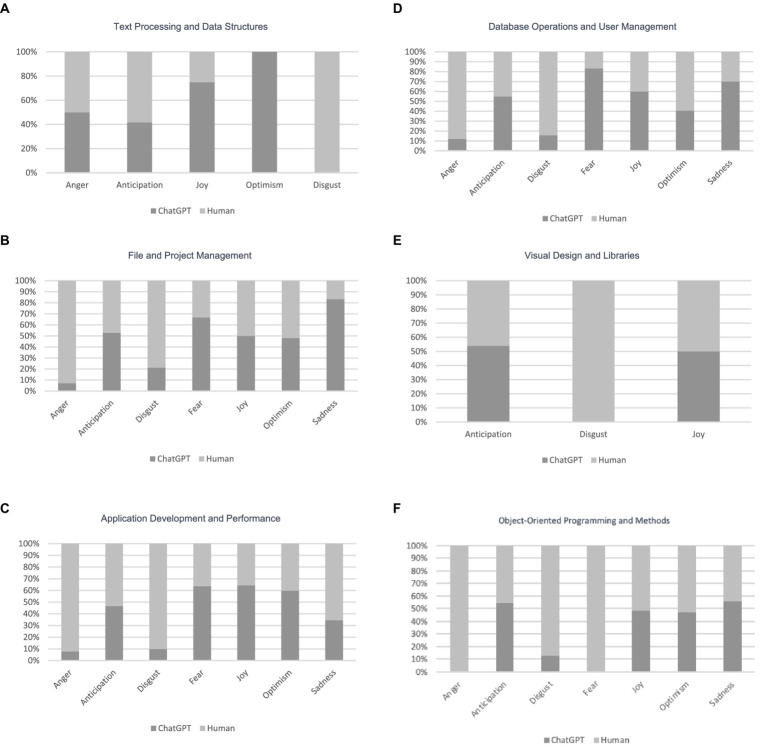
Comparison between dominant emotions of humans and ChatGPT answers for different topics.

When examining Figure 5, it becomes evident that each topic is associated with a predominant emotion. For instance, in the case of Text Processing and Data Structures topic, Disgust holds a prominent proportion compared to other emotions in the human answers.

We conducted a statistical analysis, and the results are shown in Table 5. The results confirm that in each topic, the Chi-square test results show significant differences in the proportions of emotions between ChatGPT and human responses. The very low *p*-values across all topics confirm that the observed differences are statistically significant, indicating real and substantial differences in how emotions are represented by ChatGPT versus humans in each topic (Tables 6, 7).

**Table 5:** Chi-square test results for emotional proportions in ChatGPT vs. human responses across topics.

Topic	Chi
Text Processing and Data Structures	*X^2^* (df = 6) = 324.70, *p* < 0.001
File and Project Management	*X^2^* (df = 6) = 161.30, *p* < 0.001
Application Development and Performance	*X^2^* (df = 6) = 146.39, *p* < 0.001
Database Operations and User Management	*X^2^* (df = 6) = 173.86, *p* < 0.001
Visual Design and Libraries	*X^2^* (df = 6) = 78.62, *p* < 0.001
Object Oriented Programming and Methods	*X^2^* (df = 6) = 184.33, *p* < 0.001

**Table 6:** Examples of ChatGPT answers.

Answer	Extracted emotion
Exposing the ‘eval()’ function in JavaScript to execute dynamically generated JavaScript can indeed pose a security risk. This is because any JavaScript code passed to the ‘doScript()’ function will be executed without any validation or sandboxing. By allowing the execution of raw JavaScript code, you are essentially opening up your application to potential script injection attacks, where an attacker could execute harmful or unauthorized actions on the client-side. To mitigate this risk, it is recommended to carefully validate and sanitize any user-generated JavaScript code before executing it. One approach is to leverage server-side validation and only allow specific, pre-defined operations to be executed. Alternatively, you could use a more secure method such as using ExternalInterface to call pre-defined, specific JavaScript functions that perform the necessary actions. This way, you can have more control over the execution and ensure that only trusted operations are performed. In summary, executing raw JavaScript code from Flex can be a security risk if not properly validated and sanitized. It is important to carefully consider the potential risks and implement appropriate security measures to protect your application and users.	Fear
Unfortunately, I could not find any free Vi add-ins specifically for MS Visual Studio 2005. The Google Summer of Code project you mentioned may have been promising, but it’s unclear if it has been completed or if the add-in is available. ViEmu is a popular commercial option for using Vi/Vim keybindings in Visual Studio. However, since you mentioned that you cannot test it at work and do not have a Windows computer at home, it may not be a viable solution for you. In situations like this, where you have specific requirements or restrictions, it’s always best to consult with your IT department or adhere to the company’s policy on software installation. They may provide alternative solutions or guidance on how to proceed.	Sadness
While Adhearsion is a popular choice for Ruby developers working with Asterisk, there are alternatives available for developing AGI-based IVR applications. One such alternative is the AsteriskRuby framework, which is well-documented and supported by the community. However, if you are open to considering non-Ruby frameworks, some other options include: FastAGI: FastAGI is an interface for writing AGI scripts in any programming language. It provides a simple socket-based protocol to communicate with Asterisk. This allows you to choose the programming language you are most comfortable with. PHPAGI: PHPAGI is a PHP framework specifically designed for building AGI scripts. It provides a high-level object-oriented API for working with the various features of Asterisk. Java AGI: Java AGI is a Java library for building AGI scripts. It provides a programming interface that abstracts the complexities of the AGI protocol, making it easier to develop robust IVR applications in Java. Ultimately, the choice of framework depends on your specific requirements and the programming language you are most comfortable with. It is recommended to evaluate each option, considering factors like documentation, community support, and the ease of integration with your existing infrastructure.	Optimism

**Table 7:** Examples of human answers.

Answer	Extracted emotion
SipX is really the wrong answer. I’ve written some extremely complicated VoiceXML on SipX 3.10.2 and it’s been all for naught since SipX 4 is dropping SipXVXML for an interface that requires IVRs to be compiled JARs. Top that off with Nortel filing bankruptcy, extremely poor documentation on the open-source version, poor compliance with VXML 2.0 (as of 3.10.2) and SIP standards (as of 3.10.2, does not trunk well with ITSPs). I will applaud it for a bangup job doing what it was designed to do, be a PBX. But as an IVR, if I had it to do all over again, I’d do something different. I do not know what for sure, but something different. I’m toying with Trixbox CE now and working on tying it into JVoiceXML or VoiceGlue. Also, do not read that SipX wiki crap. It compares SipX 3.10 to AsteriskNOW 1 to Trixbox 1. Come on. It’s like comparing Mac OS X to Win95! A more realistic comparison would be SipX 4 (due out 1Q 2009) to Asterisk 1.6 and Trixbox 2.6, which would show that they accomplish near identical results except in the arena of scalibility and high availability; SipX wins at that. But, for maturity and stability, I’d advocate Asterisk. Also, my real-world performance results with SipXVXML: Dell PowerEdge R200, Xeon Dual Core 3.2GHz, handles 17 calls before jitters. HP DL380 G4, Dual Xeon HT 3.2 GHz, handles 30 calles before long pauses. I’ll post my findings when I finish evaluating VoiceGlue and JVoiceXML but I think I’m going to end up writing a custom PHP called from AGI since all the tools are native to Asterisk.	Anger
ViEmu works great with Visual Studio. I used Vi(m) strictly in Linux, but I was turned on to bring the Vi(m) editing process into the Windows world by JP Boodhoo. JP praises about it also.	Joy

## 5 Discussion

We conducted a comprehensive analysis comparing emotional aspects in answers from ChatGPT and humans to 2000 questions sourced from Stack Overflow. Our findings indicate notable differences in emotional expression between humans and ChatGPT across various topics. Humans tend to have more negative responses with higher variance compared to ChatGPT. ChatGPT’s responses tend to lean toward optimism, whereas humans are more inclined toward expressing anger and disgust. This difference may be one of reasons people prefer ChatGPT’s answers 40% of the time due to their thoroughness and articulate language style (Liu et al., 2023).

When humans express an emotion, the variance is larger than in ChatGPT. It appears that ChatGPT provides responses based on patterns in training data and aims to be helpful. Human responders on Stack Overflow are real individuals with their own feelings, thoughts, and experiences.

Upon investigating the results comparing emotions in different topics, it is evident that humans tend to exhibit consistent emotional responses, encompassing feelings of Disgust or Anger. In contrast, ChatGPT demonstrates a discrepancy in emotional expression across various topics, expressing a range of emotions including Optimism, Sadness, Joy, Fear, and Anticipation. This suggests that human responses may be more authentic and natural, stemming from the inherent frustration of the searching process.

In exploring **RQ1**, the initial analysis shows that ChatGPT’s answers tend to be more optimistic and joyful, while human answers often contain more expressions of anger and disgust.

In addressing **RQ2**, when we focus on the predominant emotion and determine the maximum emotion intensity for each answer, Anticipation emerges as the dominant emotion for both the questions and the answers from humans and ChatGPT. Additionally, the results show considerable differences between ChatGPT and human responses for some emotions, such as Anger, Disgust, and Joy. Human emotions exhibit higher variance compared to ChatGPT.

Regarding **RQ3**, investigating emotions across different subjects reveals substantial differences in how emotions are represented by ChatGPT versus humans in each topic. Specifically, our analysis highlights that humans tend to exhibit a narrower emotional range within each topic, predominantly showing negative emotions like Disgust and Anger. This consistency in human emotional expression across topics could be attributed to the personal and situational frustrations users encounter when seeking help on technical issues.

Understanding these differences in emotional expression is crucial for improving AI systems like ChatGPT and holds importance from three perspectives.

Detection of AI-generated Text: The emotional differences can serve as unique fingerprints, enabling the detection of text generated by ChatGPT when compared to human-generated content.

Application in Supportive Roles: Generative AI and LLM-based chatbots may be particularly suited for roles where being a patient, optimistic, and joyful partner in dialog is beneficial, such as in education, online help, and customer service applications.

Enhancing AI Emotional Diversity: For developing more believable and empathetic AI chatbots, it is important to enhance their emotional diversity and variance. This can lead to more realistic emotional responses, making AI interactions more engaging and relatable. Future research could explore personalized and emotionally adaptive AI chatbots that reflect and respond to the user’s emotional tone. Such chatbots could be invaluable in areas like mental health counseling, child, and elderly care.

## 6 Conclusion

In this research, we conducted a comprehensive analysis comparing the emotional aspects of answers from ChatGPT and humans to 2,000 questions sourced from Stack Overflow. Our findings indicate notable differences in emotional expression between humans and ChatGPT across various topics. Humans tend to have more negative responses with higher variance compared to ChatGPT. ChatGPT’s responses tend to lean toward optimism, whereas humans are more inclined toward expressing anger and disgust.

Theses analysis highlights distinct emotional patterns across different topics. These insights underscore the need for improving AI systems to enhance their believability and user engagement, particularly in roles requiring supportive and patient interaction.

## Data Availability

The original contributions presented in the study are included in the article/supplementary material, further inquiries can be directed to the corresponding author.

## Footnotes

^1^ https://github.com/SamiaKabir/ChatGPT-Answers-to-SO-questions

## References

[ref1] Adam M. Wessel M. Benlian A. (2021). AI-based chatbots in customer service and their effects on user compliance. Electron. Mark. 31, 427–445. doi: 10.1007/s12525-020-00414-7

[ref2] Adamopoulou E. Moussiades L., (2020). “An overview of chatbot technology.” In *IFIP international conference on artificial intelligence applications and innovations* (pp. 373–383). Springer, Cham.

[ref3] Alghamdi R. Alfalqi K. (2015). A survey of topic modeling in text mining. Int. J. Adv. Comput. Sci. Appl.(IJACSA) 6, 147–153. doi: 10.14569/IJACSA.2015.060121

[ref4] Alshahrani A. (2023). The impact of ChatGPT on blended learning: current trends and future research directions. Int J Data and Network Sci 7, 2029–2040. doi: 10.5267/j.ijdns.2023.6.010

[ref5] Blei D. M. Ng A. Y. Jordan M. I. (2003). Latent dirichlet allocation. J. Mach. Learn. Res. 3, 993–1022.

[ref6] Bohn-Gettler C. M. Kaakinen J. K. (2022). Introduction to the special issue on emotions in reading, learning, and communication. Discourse Process. 59, 1–12.

[ref7] Borwankar S. Khern-am-nuai W. Kannan K. N., (2023). Unraveling the impact: An empirical investigation of ChatGPT’s exclusion from stack overflow.

[ref8] Choi J. H. Hickman K. E. Monahan A. B. Schwarcz D. (2021). ChatGPT goes to law school. J. Leg. Educ. 71:387.

[ref9] Delile Z. Radel S. Godinez J. Engstrom G. Brucker T. Young K.. (2023). “Evaluating privacy questions from stack overflow: can ChatGPT compete?.” In *2023 IEEE 31st international requirements engineering conference workshops (REW)* (pp. 239–244). IEEE.

[ref10] Devlin J. Chang M. W. Lee K. Toutanova K. (2018). Bert: pre-training of deep bidirectional transformers for language understanding. arXiv preprint arXiv 1810:04805.

[ref11] Dibble M. (2023). Schools ban ChatGPT amid fears of artificial intelligence-assisted cheating. Washington, DC: VOA News.

[ref12] Ekman P. (1992). An argument for basic emotions. Cognit. Emot. 6, 169–200.

[ref13] Elyoseph Z. Hadar-Shoval D. Asraf K. Lvovsky M. (2023). ChatGPT outperforms humans in emotional awareness evaluations. Front. Psychol. 14:1199058. 37303897 10.3389/fpsyg.2023.1199058 PMC10254409

[ref14] Frijda N. H. (2017). The laws of emotion. New York, NY: Psychology Press.

[ref15] Grudin J. Jacques R., (2019). “Chatbots, humbots, and the quest for artificial general intelligence.” In *Proceedings of the 2019 CHI conference on human factors in computing systems* (pp. 1–11).

[ref16] Hökkä P. Vähäsantanen K. Paloniemi S. (2020). Emotions in learning at work: a literature review. Vocat. Learn. 13, 1–25. doi: 10.1007/s12186-019-09226-z

[ref17] Jalil S. Rafi S. LaToza T. D. Moran K. Lam W. (2023). “Chatgpt and software testing education: promises & perils.” In *2023 IEEE international conference on software testing, verification and validation workshops (ICSTW)* (pp. 4130–4137). IEEE.

[ref18] Kabir S. Udo-Imeh D. N. Kou B. Zhang T. (2023). Who answers it better? An in-depth analysis of chatgpt and stack overflow answers to software engineering questions. arXiv preprint arXiv 2308:02312.

[ref19] Kalla D. Smith N. Samaah F. Kuraku S. (2023). Study and analysis of chat GPT and its impact on different fields of study. Int J Innovative Sci Res Technol 8, 1–12.

[ref20] Kasneci E. Seßler K. Küchemann S. Bannert M. Dementieva D. Fischer F.. (2023). ChatGPT for good? On opportunities and challenges of large language models for education. Learn. Individ. Differ. 103:102274.

[ref21] Khoury R. Avila A. R. Brunelle J. Camara B. M., (2023). “How secure is code generated by chatgpt?.” In *2023 IEEE international conference on systems, man, and cybernetics (SMC)* (pp. 2445–2451). IEEE.

[ref22] Kung T. H. Cheatham M. Medenilla A. Sillos C. De Leon L. Elepaño C.. (2023). Performance of ChatGPT on USMLE: potential for AI-assisted medical education using large language models. PLoS Digital Health 2:e0000198. doi: 10.1371/journal.pdig.0000198, PMID: 36812645 PMC9931230

[ref23] Li J. Xiao L., (2023). “Multi-emotion recognition using multi-EmoBERT and emotion analysis in fake news.” In *Proceedings of the 15th ACM web science conference 2023* (pp. 128–135).

[ref24] Liu J. Tang X. Li L. Chen P. Liu Y. (2023). Which is a better programming assistant? A comparative study between chatgpt and stack overflow. arXiv preprint arXiv 2308:13851.

[ref25] Mhlanga D. (2023). “Open AI in education, the responsible and ethical use of ChatGPT towards lifelong learning” in FinTech and artificial intelligence for sustainable development: The role of smart technologies in achieving development goals (Springer Nature Switzerland: Cham), 387–409.

[ref26] Mohammad S. Bravo-Marquez F. Salameh M. Kiritchenko S. (2018). “Semeval-2018 task 1: affect in tweets.” In *Proceedings of the 12th international workshop on semantic evaluation* (pp. 1–17).

[ref27] Murad I. A. Surameery N. M. S. Shakor M. Y. (2023). Adopting ChatGPT to enhance educational experiences. Int J Info Technol Comp Eng 3, 20–25. doi: 10.55529/ijitc.35.20.25

[ref28] Nandrino J. L. Baracca M. Antoine P. Paget V. Bydlowski S. Carton S. (2013). Level of emotional awareness in the general French population: effects of gender, age, and education level. Int. J. Psychol. 48, 1072–1079. doi: 10.1080/00207594.2012.753149, PMID: 23305070

[ref29] Nascimento N. Alencar P. Cowan D. (2023). Comparing software developers with chatgpt: an empirical investigation. arXiv preprint arXiv 2305:11837.

[ref30] Okey O. D. Udo E. U. Rosa R. L. Rodríguez D. Z. Kleinschmidt J. H. (2023). Investigating ChatGPT and cybersecurity: a perspective on topic modeling and sentiment analysis. Comput. Secur. 135:103476. doi: 10.1016/j.cose.2023.103476

[ref31] Open AI. (2023). “ChatGPT.” Available at: https://openai.com/blog/chatgpt

[ref32] Ouyang L. Wu J. Jiang X. Almeida D. Wainwright C. Mishkin P.. (2022). Training language models to follow instructions with human feedback. Adv. Neural Inf. Proces. Syst. 35, 27730–27744.

[ref33] Pamungkas E. W. (2019). Emotionally-aware chatbots: a survey. arXiv preprint arXiv 1906:09774.

[ref34] Parker J. L. Becker K. Carroca C. (2023). ChatGPT for automated writing evaluation in scholarly writing instruction. J. Nurs. Educ. 62, 721–727. doi: 10.3928/01484834-20231006-02, PMID: 38049299

[ref35] Parrott W. G. (2001). Emotions in social psychology: Essential readings. New York, NY, US: psychology press.

[ref36] Plutchik R. (1980). “A general psychoevolutionary theory of emotion” in Theories of emotion (New York: Academic press), 3–33.

[ref37] Poremba S., (2023). ChatGPT confirms data breach, raising security concerns. Retrieved from security intelligence website: https://securityintelligence.com/articles/chatgpt-confirms-data-breach.

[ref38] Rapp A. Curti L. Boldi A. (2021). The human side of human-chatbot interaction: a systematic literature review of ten years of research on text-based chatbots. Int J Human-Computer Stud 151:102630. doi: 10.1016/j.ijhcs.2021.102630

[ref39] Russell J. A. (1980). A circumplex model of affect. J. Pers. Soc. Psychol. 39:1161. doi: 10.1037/h0077714, PMID: 38605847

[ref40] Saini N. (2023). ChatGPT becomes fastest growing app in the world, records 100mn users in 2 month. LiveMint.

[ref41] Spolsky J. Atwood J., (2008). Introducing stack overflow.

[ref42] Surameery N. M. S. Shakor M. Y. (2023). Use chat GPT to solve programming bugs. Int J Info Technol Comp Eng (IJITC) 3, 17–22. doi: 10.55529/ijitc.31.17.22

[ref43] Susnjak T. McIntosh T. R. (2024). ChatGPT: the end of online exam integrity? Educ. Sci. 14:656. doi: 10.3390/educsci14060656

[ref44] Tyng C. M. Amin H. U. Saad M. N. Malik A. S. (2017). The influences of emotion on learning and memory. Front. Psychol. 8:235933. doi: 10.3389/fpsyg.2017.01454, PMID: 28883804 PMC5573739

[ref45] Um E. Plass J. L. Hayward E. O. Homer B. D. (2012). Emotional design in multimedia learning. J. Educ. Psychol. 104:485. doi: 10.1037/a0026609, PMID: 38923742

[ref46] Wang X. Pang H. Wallace M. P. Wang Q. Chen W. (2024). Learners’ perceived AI presences in AI-supported language learning: a study of AI as a humanized agent from community of inquiry. Comput. Assist. Lang. Learn. 37, 814–840. doi: 10.1080/09588221.2022.2056203

[ref47] Malwarebytes (2023). What is ChatGPT? ChatGPT Security Risks. Available at: https://www.malwarebytes.com/cybersecurity/basics/chatgpt-ai-security (Accessed on Jun. 26, 2023)

[ref48] Zhao W. Huang S. Yan L., (2024). “ChatGPT and the future of translators: overview of the application of interactive AI in English translation teaching.” In *2024 4th international conference on computer communication and artificial intelligence (CCAI)* (pp. 303–307). IEEE.

